# Keep Calm and Survive: Adaptation Strategies to Energy Crisis in Fruit Trees under Root Hypoxia

**DOI:** 10.3390/plants9091108

**Published:** 2020-08-27

**Authors:** Ariel Salvatierra, Guillermo Toro, Patricio Mateluna, Ismael Opazo, Mauricio Ortiz, Paula Pimentel

**Affiliations:** 1 Plant Genomics Laboratory, Centro de Estudios Avanzados en Fruticultura (CEAF), Rengo 2940000, Chile; asalvatierra@ceaf.cl; 2 Plant Stress Physiology Laboratory, Centro de Estudios Avanzados en Fruticultura (CEAF), Rengo 2940000, Chile; gtoro@ceaf.cl (G.T.); pmateluna@ceaf.cl (P.M.); 3 Plant Breeding Laboratory, Centro de Estudios Avanzados en Fruticultura (CEAF), Rengo 2940000, Chile; iopazo@ceaf.cl; 4 Agronomy Laboratory, Centro de Estudios Avanzados en Fruticultura (CEAF), Rengo 2940000, Chile; mortiz@ceaf.cl

**Keywords:** hypoxia, waterlogging, fruit trees, *Prunus*, aerenchyma, hypertrophied lenticels, anaerobic fermentation, energy metabolism, root respiration

## Abstract

Plants are permanently facing challenges imposed by the environment which, in the context of the current scenario of global climate change, implies a constant process of adaptation to survive and even, in the case of crops, at least maintain yield. O_2_ deficiency at the rhizosphere level, i.e., root hypoxia, is one of the factors with the greatest impact at whole-plant level. At cellular level, this O_2_ deficiency provokes a disturbance in the energy metabolism which has notable consequences on the yield of plant crops. In this sense, although several physiological studies describe processes involved in plant adaptation to root hypoxia in woody fruit trees, with emphasis on the negative impacts on photosynthetic rate, there are very few studies that include -omics strategies for specifically understanding these processes in the roots of such species. Through a de novo assembly approach, a comparative transcriptome study of waterlogged *Prunus* spp. genotypes contrasting in their tolerance to root hypoxia was revisited in order to gain a deeper insight into the reconfiguration of pivotal pathways involved in energy metabolism. This re-analysis describes the classically altered pathways seen in the roots of woody fruit trees under hypoxia, but also routes that link them to pathways involved with nitrogen assimilation and the maintenance of cytoplasmic pH and glycolytic flow. In addition, the effects of root hypoxia on the transcription of genes related to the mitochondrial oxidative phosphorylation system, responsible for providing adenosine triphosphate (ATP) to the cell, are discussed in terms of their roles in the energy balance, reactive oxygen species (ROS) metabolism and aerenchyma formation. This review compiles key findings that help to explain the trait of tolerance to root hypoxia in woody fruit species, giving special attention to their strategies for managing the energy crisis. Finally, research challenges addressing less-explored topics in recovery and stress memory in woody fruit trees are pointed out.

## 1. Introduction

Plants are aerobic organisms and sensitive to many external conditions that could alter internal homeostasis. O_2_ deficiency or deprivation trigger hypoxia or anoxia stress, respectively, depending on the O_2_ concentrations. Plants can face hypoxic conditions from different origins: developmental hypoxia, in plant–microbe interactions, or environmental hypoxia (waterlogging/submergence) [1].

Currently, the cultivation surface of fruit trees in Mediterranean and subtropical areas is approximately 49 million hectares. Of this group, 22% corresponds to olive trees, 20% to citrus, 16% to stone fruit, 15% to vines and 13% to pome fruit [2]. Fruit trees of the Mediterranean climate, such as walnut (*Juglans regia* L.), apple (*Malus sylvestris* L.) or sweet cherry (*Prunus avium* L.), show a low tolerance to hypoxia compared to trees from wetland areas [3]. In addition, most of these fruit trees do not have the capacity for acclimatization, or even for the recovery of their physiological and growth parameters while flooded, unlike tree forest species of the wetland areas [4,5,6]. However, several studies have described some hypoxia–tolerance gradient among fruit trees [4,5,6,7].

In the context of fruit production, rootstocks are selected for rooting and grafting capacity, abiotic and biotic stress tolerance, and their ability to beneficially alter scion phenotypes, especially in yielding terms. In perennial and some vegetable crops, grafting is used to join resilient root systems (rootstocks) to shoots (scions) that produce the harvested product [8]. The hydraulic architecture of rootstocks becomes of fundamental importance, since the sustained flow of water controls many plant processes, such as growth, mineral nutrition, scion photosynthesis and transpiration [9]. Species of *Prunus* used as rootstocks are classified as moderately sensitive to root hypoxia, although differences among genotypes regarding their ability to tolerate this stress have been reported [7,10,11,12,13,14]

Mediterranean agriculture has coevolved with harsh environments and changing climate conditions over millennia, generating an extremely rich heritage, but actual climatic change threatens global agriculture especially given the prevalence of highly specialized, low diverse agroecosystems [15]. The impact of global warming plus a human population hovering around 7.7 billion strongly challenges agricultural systems, and the need for a better understanding of how crops and fruit trees can survive and yield under more extreme climatic events becomes of paramount importance, since an increase in precipitation intensity and variability would increase the risks of flooding [16].

Nowadays, as a consequence of global climate change, there are areas of the planet most exposed to intense rains where accumulated amount of water can lead to soil saturation, with a concomitant displacement of O_2_ in the rhizosphere. However, although less discussed, it is important to note that there are a number of conditions, beyond excess rainfall, that can configure the establishment of O_2_ deficiency at the root level.

## 2. Edaphic Conditions that Promote O_2_ Deficiency

The air capacity of the soil is determined by its texture and structure. Coarse-textured soils can have an air capacity of around 25%, while in fine-textured soils the air capacity can reach 10% [17]. However, when the structure is altered by physicochemical processes or mechanical forces, the macroscopic pores tend to disappear, so a strongly compacted soil may contain less than 5% air by volume at its characteristic field-capacity value of soil moisture [17].

Fine texture and compaction associated with bad irrigation practices are able to generate an excess of water sufficient for establishing an O_2_ deficit at rhizosphere level. Silt and clay particles reduce soil aeration because they are tightly packed together, decreasing the air spaces between them and slowing the drainage [18]. This condition creates an O_2_ deficient environment for plants by maintaining high moisture on the soil surface [19]. In fine-textured soils, transient soil waterlogging can be generated only by poor irrigation management [20]. Soil compaction occurs primarily when pressure exerted on the soil surface reduces the air spaces between soil particles, and it is associated with agricultural practices, but it can also be the result of natural processes unrelated to the application of compressive forces [19]. This results in a change in the proportion of pores with water and air (mainly loss of coarse pores), and an increase in mechanical resistance to root development [19,21,22]. The soil compaction may be superficial or even reach 100 mm or more [19], which could have an important impact on the perennial fruit tree’s development. In almond, a field experiment was performed using a heavy tractor to evaluate the continuous transit of agricultural machinery over the soil simulating multiple applications. The authors concluded that the continuous heavy tractor traffic causes an evident soil compaction (20–40 cm deep) with a drastic decrease in soil porosity reaching up to 11%, and it also produced an increase in bulk density and cone index in subsoil layers [23].

Plants need an adequate supply of air and water in the soil pore space for their suitable development and growth [24]. The rate of the transfer of gases in the air phase is generally much greater than in the water phase; hence soil aeration depends largely on the volume fraction of air-filled pores [17]. Typically, the requirement for plant development is for at least 10% of the soil volume to comprise gas-filled pores at field capacity (air capacity), and for at least 10% of the gas in these pores to be O_2_ [25]. Cook and Knight [24] determined that when the air-filled porosity of the soil drops below 12%, the root respiration and the exchange of O_2_ and CO_2_ between the soil and the atmosphere is hampered. Kawase [26] points out that when it drops below 10%, hypoxia is triggered, and under more drastic conditions it becomes in anoxia.

Hypoxia/anoxia conditions restrict processes such as plant respiration, water and nutrient absorption. Anaerobic conditions in the soil induce a series of physical, chemical and biological processes, such as pH and redox potential, resulting in changes to the soil’s elemental profile [27]. O_2_ deficiency further affects microbial communities and microbial processes in the soil [28]. Once free O_2_ is consumed, nitrate is used by soil microorganisms as an alternative electron acceptor to continue their respiration. The following acceptors are MnO_2_, Fe(OH)_3_, SO_4_^2−^ and CO_2_ [29]. This generates an increase in the levels of reduced compounds, such as Mn^2+^, Fe^2+^, H_2_S, NH_4_^+^ and organic compounds (alkanes, acids, carbonyls, etc.) [30,31]. These solutes can accumulate up to phytotoxic levels and contribute to plant injury [32].

## 3. Fruit Tree Responses to O_2_ Deficiency

### 3.1. Physiological and Biochemical Response of Fruit Trees under O_2_ Deficiency

Under hypoxia stress, it has been observed that the gas exchange parameters are dramatically affected in several fruit trees, such as avocado (*Persea americana* Mill.) [33], kiwi fruits (*Actinidia chinensis* Planch) [34], citrus trees [35,36,37,38], pecans (*Carya illinoensis* K. Koch) [39], walnut trees (*Juglans regia* L.) [40], grapevine (*Vitis vinifera* L.) [41,42], pomegranate (*Punica granatum* L.) [43], apple (*Malus* × *domestica* Borkh) [44] and several *Prunus* species [7,45,46,47,48,49]. In general, all these tree species were classified as sensitive to root hypoxia. However, the concept of “relative tolerance” to hypoxia applied to the fruit tree species must be viewed with caution as many factors, such climatic, experimental or edaphic conditions, among others, could influence the responses observed [50]. However, some classifications have been made: extremely tolerant—quince (*Cydonia oblonga* Mill.) and *Pyrus betulaefolia*; very tolerant—pear (*Pyrus* spp.); moderately tolerant—apple (*Malus* × *domestica* Borkh), *Citrus* spp., and plums (*Prunus domestica* L. and *Prunus cerasifera* Ehrh); moderately sensitive—plum (*Prunus salicina* Lind.); very sensitive—cherry (*Prunus avium* L); extremely sensitive, peach (*Prunus persica* Batsch); and most sensitive—almond (*Prunus dulcis* [Mill.] DA Webb) and apricot (*Prunus armeniaca* L.) [50]. In the particular case of *Prunus* species, a tolerance gradient to long-term hypoxia was reported among seven genotypes used as rootstocks, identifying as tolerant to ‘Mariana 2624’ (*Prunus cerasifera* × *Prunus munsoniana* W. Wight and Hedrick) a plum rootstock, and as the most sensitive to ‘Mazzard F12/1’ (*P. avium*) a cherry rootstock [7]. ‘Mariana 2624’ plants survived through 14 days of waterlogging treatment, showing similar stomatal conductance and CO_2_ assimilation rate values between waterlogged and control plants, unlike in the hypoxia-sensitive genotype, which showed intense leaf and root damage and a drastic decrease in the gas exchange parameters of the leaves during root hypoxia [7]. The ability to maintain a high photosynthetic rate, such as that observed in hypoxia-tolerant species, would guarantee an adequate supply of carbohydrates from the leaves to the roots. The carbohydrate supply is correlated with the production of highly energetic molecules (ATP), and the level of carbohydrate reserves or the capacity to maintain their transport throughout the plant appears to be a key feature in the tolerance to long-term flooding [48,51,52,53,54]. Consequently, maintaining glycolysis by a steady and sufficient supply with carbohydrates seems to be crucial for survival under hypoxia [3].

One of the first responses to O_2_ deprivation is a hydraulic adjustment, the purpose of which is to sustain a constant water supply from roots to shoots, which is essential to maintaining gas exchange parameters in hypoxia-tolerant species [3,55]. Root hydraulic conductance is also affected under hypoxia stress, usually decreasing this parameter, but the response would depend on the species, age and even the experimental set-up [56]. There is a huge body of literature about the importance of root water transport in plants under different abiotic stresses (reviewed in [56,57,58]). In this context, root hypoxia modifies the root water transport in different manners: (1) cellular acidosis and the depletion of ATP affect the phosphorylation of aquaporins and the transport through these water channels is inhibited [59]; (2) hypoxia can alter root structure by inducing suberization (generation of a radial oxygen loss (ROL) barrier), but at the same time, this modification can affect the apoplastic water transport [60]; and (3) massive damage of the root system [3,32]. After 15 days of long-term waterlogging, the hypoxia-tolerant genotype “Mariana 2624” showed similar values of root hydraulic conductance (*Kr*) between normoxic and waterlogged plants. Unlike these, the hypoxia-sensitive genotype showed a strong decrease in *Kr* triggered by hypoxia (Pimentel, unpublished data).

Reactive oxygen species (ROS) are by-products of various metabolic pathways and are generated enzymatically or nonenzymatically [61]. Nonenzymatic ROS production can occur in mitochondria and chloroplast through electron transport chains (ETC) [61,62,63]. Enzymatic ROS production can occur in peroxisomes, cell walls, plasma membrane and apoplast [64], and also through respiratory burst oxidase homologs (RBOHs), a plasma-membrane-bound NADPH oxidase [62,65]. ROS induce [Ca^2+^]_cyt_ elevations by activation of the specialized Ca^2+^-permeable ion channels in the plasma membrane. In addition, NADPH oxidases (RBOHs) are directly activated by cytosolic Ca^2+^. Both ROS and Ca^2+^ form a self-amplifying loop named “ROS-Ca^2+^ hub” [66]. Elevation of [Ca^2+^]_cyt_ under hypoxia triggers multiple metabolic events and it is associated with both early and late responses to low oxygen conditions (deeply reviewed in [67]). ROS generated in response to abiotic stresses may be involved in various responses, acting as signaling molecules or triggering ROS-induced cell death [65]. Under hypoxic stress conditions, ROS can be generated due to an impairment of photosynthesis and aerobic respiration processes by inhibiting mETC [54,61,68]. In some cases, re-oxygenation of the soil after prolonged flooding can cause severe oxidative damage to the roots of sensitive trees [36,69]. Indeed, re-oxygenation has been recognized as an abiotic stress that can injure plants post-submergence (reviewed in [36,69])

In a re-analysis of the transcriptome published by Arismendi et al. [70] (commented on in Section 4), it was possible to find three differentially expressed isoforms of the *RBOH* gene, *RBOHA*, *RBOHC* and *RBOHE*. *RBOHA* and *RBOHE* genes showed a similar pattern between the two rootstocks, being that both genes were upregulated in hypoxic conditions. On the other hand, the *RBOHC* gene was downregulated in the hypoxia-tolerant genotype ‘Mariana 2624,’ but induced in the sensitive one under hypoxia stress (Table 1). Interestingly, the *RBOHC* gene has been reported as principally expressed in roots, where it is related to root hair formation and primary root elongation and development in Arabidopsis [65]. ROS have been described as toxic molecules generated by aerobic respiration that can cause oxidative damage. However, ROS also play a key role in signaling to trigger several processes such as cell proliferation and differentiation [71]. The hypoxia-sensitive genotype response suggests an ROS signaling role in the early stages of O_2_ deficiency. Thus, ‘Mazzard F12/1’ could activate the formation of new roots, possibly replacing the original root system progressively injured in hypoxia.

As ROS accumulates after hypoxic events, the probability of the cell membrane being involved in a lipoperoxidation process is increased. Malondialdehyde (MDA) and electrolyte leakage are widely used as indicators of oxidative damage in plants. Differences in the accumulation of MDA have been reported under hypoxia, which depend on the degree of tolerance of the genotypes evaluated. For instance, in citrus species, the most tolerant genotype showed a delayed accumulation of MDA in leaves and roots in comparison with the other genotypes under hypoxia conditions [72]. Hypoxia treatment dramatically increased MDA content in the roots of two *Malus* species, but higher concentrations of H_2_O_2_ and superoxide radicals (O_2_^•–^) were detected in the hypoxia-sensitive species [73]. In *Prunus* rootstocks under long-term hypoxia, ‘Mariana 2624’ plants, the hypoxia-tolerant genotype, showed no significant changes in MDA concentration in roots and leaves in comparison with the control plants. Opposingly, the sensitive rootstock ‘Mazzard F12/1’ showed a higher MDA concentration in roots and leaves after seven days of waterlogging, suggesting less capacity to remove ROS than the tolerant genotype [7,47]. Along with a lower MDA content in the roots of the hypoxia-tolerant genotype, a lower electrolyte leakage rate was also observed. Both parameters evidenced lesser structural damage in the roots, and are related to higher root membrane stability in ‘Mariana 2624’ [74].

Plants have enzymatic and non-enzymatic antioxidant compounds that participate in ROS detoxification, reducing the phytotoxic effects of radical species at the cellular level [75,76]. In citrus trees, a higher tolerance to O_2_ deficiency by flooding is associated with the ability to delay the apparition of oxidative damage caused by a high activity of antioxidant enzymes such as superoxide dismutase (SOD), ascorbate peroxidase (APX), glutathione reductase (GR) and catalase (CAT) [72]. In Citrumelo trees, the induction of SOD, APX and CAT enzyme activities allows them to maintain a transient tolerance under hypoxia [36]. Similar results were observed in *Malus* [73] and *Prunus* species under hypoxia [77]. However, different results were found by Amador et al. (2012), since an increase in CAT activity in short-term waterlogging was found in the hypoxia-sensitive hybrid ‘Felinem’, but not in the hypoxia-tolerant rootstock ‘Myrobalan’. The authors mention that they cannot conclude that antioxidant enzymes are directly involved in the tolerance of the hypoxia-tolerant genotype. The re-analysis of Arismendi et al. [70] evidenced that, in general terms, there are no notable differences in the expression of the *SOD*, *CAT* and *APX* genes between the hypoxia-sensitive and hypoxia-tolerant genotypes, except in two cases. In the first one, the *CuZnSOD2* gene showed an upregulated expression pattern in the hypoxia-tolerant genotype, and a significant downregulation in the hypoxia-sensitive one. In the second case, the *APX5* gene showed upregulation in both genotypes, but with a significantly higher expression in the tolerant one (Table 1). These results could explain the differences in MDA content and electrolyte leakage between the *Prunus* rootstocks, since the belated rise in the MDA concentration and electrolyte leakage in the root hypoxia-sensitive genotype suggests a lesser capacity to remove ROS, implying the occurrence of massive tissue damage which compromises the survival of the plant, as reported by Pimentel et al. [7] and Toro et al. [74].

### 3.2. Morpho-Anatomical Changes in Fruit Trees under O_2_ Deficiency

Roots are the first organ that directly sense and deal with O_2_ deficiency in compacted waterlogged or flooded soils. Therefore, morpho-anatomical modifications that allow the maintaining of better root oxygenation in waterlogging conditions are one of the key mechanisms associated with hypoxia-tolerant genotypes [78]. Adventitious roots, hypertrophied lenticels and aerenchyma all contribute to oxygenating the root system under low O_2_ conditions [79,80]. Adventitious roots are produced in the replacement of damaged root systems, and are usually thicker and have more intercellular gas-filled spaces than roots growing in well-aerated soil [79]. Hypertrophied lenticels and aerenchyma favor O_2_ diffusion to the root tips and rhizosphere, and in addition the hypertrophied lenticels allow the outwards diffusion of potentially toxic compounds like ethanol, acetaldehyde, ethylene and CO_2_ [5,80,81]. In annual crops, both hypertrophied lenticels and the formation of aerenchyma generate a snorkel effect that keeps the roots oxygenated under waterlogging conditions [82], and eliminate toxic products generated from lactic and ethanol fermentation [79,80]. Another anatomical modification in roots is the development of the radial oxygen loss (ROL) barrier observed in many wetland plants. This barrier promotes longitudinal O_2_ diffusion down roots, restricts the O_2_ loss to the soil and could reduce the entry of phytotoxins into the roots in waterlogged soils [83,84,85,86,87]. This root trait has been not identified or studied in hypoxia-tolerant woody and perennial upland fruit trees yet.

The generation of morpho-anatomical modifications has been observed in different fruit tree species. Hypoxia-tolerant apple rootstocks generated adventitious roots in response to intermittent waterlogging events [88]. The development of hypertrophied lenticels as a response to root hypoxia has been reported in Rosaceae species such as *Pyrus* spp. and *Cydonia oblonga* (Mill.) [79]. Pistelli et al. [52] reported the formation of adventitious roots in *Prunus cerasifera* L. (‘Mr.S.2/5_rootstock’) under waterlogging stress, but they did not report the development of aerenchyma. Pimentel et al. [7] reported that the hypoxia-tolerant *Prunus* rootstock ‘Mariana 2624’ was able to develop adventitious roots, aerenchyma in adventitious roots and hypertrophied lenticels 10 days after the onset of waterlogging treatment. A large adventitious root system grew from the stem base and just beneath the water surface. In this new root system, aerenchyma tissue was not observed near the root apex, but was widely developed distant from it (at 30 and 55 mm from the root apex) and, at the same time, the development of hypertrophied lenticels on the submerged portion of stems was observed (Figure 3). Hypertrophied lenticels appear in wetland species and in several woody and herbaceous plant species subjected to waterlogging [80,89,90]. In *Prunus* rootstocks, the hypertrophied lenticels were developed only in the hypoxia-tolerant genotypes, and when the submerged stems of the rootstock ‘Mariana 2624’ were sealed with lanoline to prevent its development, a significant decrease in gas exchange parameters was detected in comparison with the non-lanoline-treated plants [7]. Interestingly, Toro et al. [74] showed that aerenchyma formation also takes place in the existing root system in ‘Mariana 2624’ at 30 mm from the root apex (Figure 3). In woody plants, all these morpho-anatomical modifications are late responses. In the case of long-term hypoxia treatment, they appear after 6 or 10 days from the beginning of the stress, and they are part of the strategies used to avoid the negative effects of the energetic crisis triggered by an O_2_ deficient environment.

## 4. Transcriptomic Reprogramming of Principal Pathways Involved in Energy Metabolism under O_2_ Deficiency

The effect of O_2_ deficiency on the rhizosphere of woody plant species has been more widely addressed from a physiological perspective than from a genomic point of view. Despite the massive amount of data provided by next generation sequencing analyses (NGS), which would allow a more integrative view of the plant’s response to root hypoxia stress, few transcriptomic studies have been reported in woody species [91,92,93] and fruit trees such as avocado [94] *Prunus* sp. [49,53,70,95], kiwi fruit [96] and grapevine [41,97].

Using RNA-seq time series data from our collaborative study on *Prunus* rootstocks previously reported [70], a revisited analysis with a de novo assembly approach was carried out in order to gain a deeper insight into the relevant pathways involved in energy metabolism under hypoxic conditions. The de novo assembly evidenced a higher number of differentially expressed genes (DEGs) in the hypoxia-sensitive *Prunus* rootstock ‘Mazzard F12/1’ at each of the sampling times of the waterlogging treatment, in comparison to the hypoxia-tolerant rootstock ‘Mariana 2624’ (6 h: 2638 vs. 2356; 24 h: 5819 vs. 5228 and 72 h: 10,255 vs. 6366). Specifically, those RNA-Seq analyses focused on the hypoxia adaptation of root systems [41,53,70,94,95] have evidenced certain metabolic pathways and groups of genes commonly affected by O_2_ deficiency.

**Figure 1:**
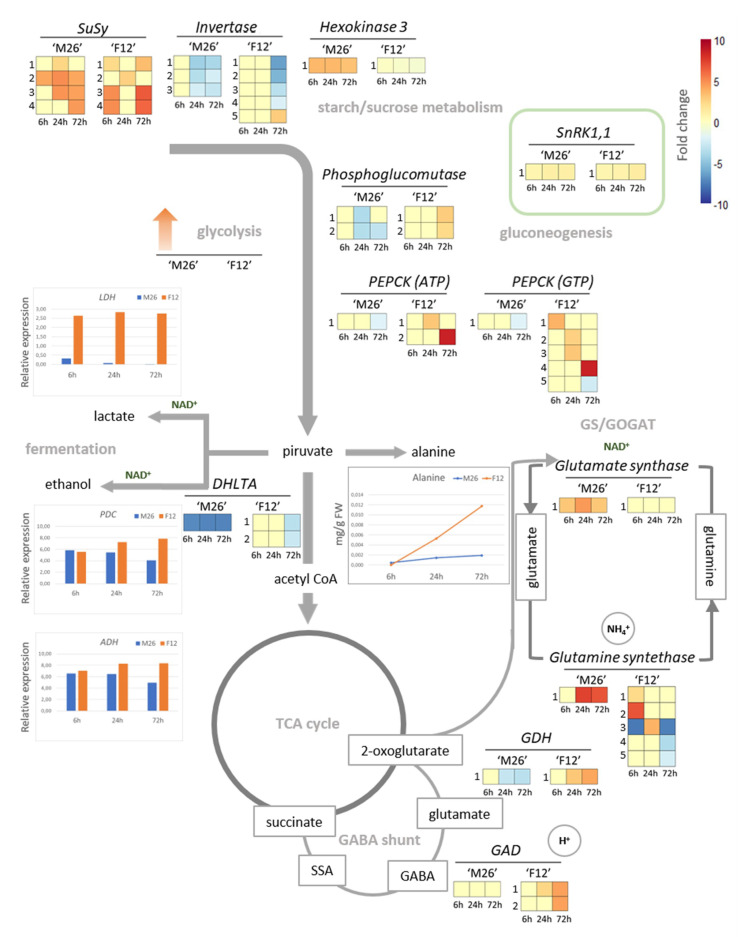
Transcriptomic reconfiguration of pathways involved in energy metabolism in response to O_2_ deficiency. Through a comparative analysis between two genotypes of *Prunus* rootstocks, the hypoxia-tolerant ‘Mariana 2624’ (‘M26′) and the hypoxia-sensitive ‘Mazzard F12/1’ (‘F12′), the changes in the transcript levels of genes belonging to the routes typically related to energy metabolism (starch/sucrose, glycolysis/gluconeogenesis and lactic/ethanolic fermentations) and that are expressed in a contrasting way between the genotypes are described. Along with these routes, their connections to TCA cycle, GABA shunt and the GS/GOGAT cycle are presented. The differential transcript levels of the genes of the last two pathways show clear differences that point to the favoring of one or another metabolic pathway in a genotype-dependent manner. In addition, the steps where the regeneration of NAD^+^, consumption of excess H^+^ (regulation of cytoplasmic pH) and assimilation of NH_4_^+^ take place are shown. The data included in this re-analysis were obtained through a de novo approach via RNAseq data retrieved from NCBI BioProjects PRJNA215360 and PRJNA215068 [70], corresponding to *P*. *cerasifera* × *P*. *munsoniana* ‘Mariana 2624’ and *P*. *avium* ‘Mazzard F12/1’, respectively. Quality control of the libraries was performed with FastQC and the AfterQC tool [102]. Consecutively, all libraries were merged into one unique file to perform a de novo assembly with Trinity [103]. The resulting fasta file was depurated to obtain unigenes using Transdecoder with the pFam database [104] and CD-HIT-EST [105,106]. This output was used as a reference to perform an alignment using Hisat2 [107]. Transcript assembly was performed through StringTie [108]. DEGs were obtained using EdgeR from Bioconductor [109]. Genes with adjusted *p*-value < 0.05 and LogFC > 2 & < −2 were used for further analysis. The annotation was performed using GO FEAT [110] and KEGG [111]. DEGs included in this figure are indicated with their homolog loci from *P*. *persica*: *SuSy—*(1) Prupe.7G192300.4; (2) Prupe.8G264300.1; (3) Prupe.1G131700.1; (4) Prupe.7G192300.1. *Invertase—*(1) Prupe.2G277900.1; (2) Prupe.2G277900.1; (3) Prupe.6G122600.1; (4) Prupe.6G122600.2; (5) Prupe.1G111800.1. *Hexokinase 3—*(1) Prupe.1G366000. *SnRK1,1—*(1) Prupe.3G262900.1. *Phosphoglucomutase—*(1) Prupe.2G286900.1; (2) Prupe.1G330700.1. *PEPCK (ATP)—*(1) Prupe.6G211000.1; (2) Prupe.1G541200.7. *PEPCK (GTP)—*(1) Prupe.1G541200.4; (2) Prupe.6G210900.1; (3) Prupe.6G211000.1; (4) Prupe.1G541200.7; (5) Prupe.4G166400.4. *DHLTA—*(1) Prupe.1G309100.1; (2) Prupe.8G056000.1. *Glutamate synthase—*(1) Prupe.2G311700.2. *Glutamine syntethase—*(1) Prupe.1G148700.1; (2) Prupe.3G166500.3; (3) Prupe.1G346600.1; (4) Prupe.5G236300.1; (5) Prupe.3G166500.2. *GDH—*(1) Prupe.7G004100.1; (2) Prupe.2G269800.1. *GAD—*(1) Prupe.1G339900.1; (2) Prupe.7G252300.1. For more detailed results of the transcriptional patterns of *LDH*, *PDC* and *ADH* (Quantitative Reverse Transcription (qRT)-PCR) and alanine levels (HPLC-DAD) inserted in this figure, refer to [112,47].

Maintaining a continuous glycolytic flux is a crucial factor in ensuring the energy pool necessary for the processes involved in the survival of trees under hypoxia [3]. In woody species, it has been reported that hypoxia-sensitive plants deplete their soluble sugars rapidly in flooded conditions, but the tolerant ones keep a higher level of soluble sugars for longer periods [81,98,99]. In this context, the regulation of gene expression involved in primary metabolism and energy homeostasis is essential to avoiding the detrimental effects of energy depletion under hypoxic stress. In the sweet cherry rootstock *Cerasus sachalinensis* (F. Schmidt), the waterlogging treatment upregulated most genes associated with sucrose metabolism. Genes encoding *SUCROSE SYNTHASE* (*SuSy*) were upregulated, however *INVERTASE* (*INV*) genes were downregulated [53]. In ‘Mariana 2624’ and ‘Mazzard F12/1’ plants, four *SuSy* isoforms were upregulated under hypoxic conditions. Additionally, in ‘Mazzard F12/1’ rootstocks, one *INV* isoform was upregulated after 72 h of waterlogging treatment, and four were downregulated at the same time. On the other hand, ‘Mariana 2624’ rootstocks exhibited three *INV* isoforms downregulated at 24 and 72 h (Figure 1). Both *SuSy* and *INV* can cleave sucrose to release its constituent monosaccharides, although a lower energy cost of generating hexose phosphates for glycolysis is required in the case of sucrose synthase. This latter is a typical feature of sucrose metabolism during O_2_ deficiency [100]. Thus, those plants that favor the activity of SuSy would be opting for a more energy efficient way to provide substrates for glycolysis under hypoxic conditions.

Transcriptomic evidences in the roots of waterlogged forestry trees revealed enhanced glycolytic flux and an activation of fermentative pathways in order to maintain the energy supply when mitochondrial respiration is inhibited by O_2_ deficiency [81,91]. Alongside the induction of genes related to glycolysis, an absence of transcripts for genes associated to gluconeogenesis, such as *GLUCOSE 6-PHOSPHATASE* and *FRUCTOSE 1,6-BISPHOSPHATASE*, was reported in roots of flooded avocado (*Persea americana* Mill.) [94]. In this sense, the downregulation of *PHOSPHOGLUCOMUTASE* genes reported in flooded grapevine roots also supports the idea of a hampered flux to gluconeogenesis under O_2_ deficiency [41]. This pattern was also presented in Myrobalan ‘P.2175’, another hypoxia-tolerant *Prunus* rootstock, but not in the hypoxia-sensitive ‘Felinem’ [95]. In the *Prunus* rootstock ‘Mariana 2624’, two *PHOSPHOGLUCOMUTASE* isoforms were downregulated at 24 and 72 h of waterlogging treatment, but the gene induction of two isoforms detected in ‘Mazzard F12/1’ plants after 72 h of O_2_ deficiency is worth noting (Figure 1). Furthermore, genes encoding for *PHOSPHOENOLPYRUVATE CARBOXYKINASES* (*PEPCK*) repeated the behavior described for *PHOSPHOGLUCOMUTASE* in these *Prunus* rootstocks contrasting in their tolerance to hypoxia (Figure 1). Regarding the above-mentioned, the inhibition of gluconeogenesis appears to be strongly related to hypoxia-tolerant genotypes in *Prunus* spp.

HEXOSE-PHOSPHORYLATING HEXOKINASES (HXK) are the only plant enzymes able to phosphorylate glucose, so they are considered a key factor in glycolysis activation [101]. The hypoxia-tolerant *Prunus* rootstock ‘Mariana 2624’ evidenced the induction of more *HXK* genes than the hypoxia-sensitive ‘Mazzard F12/1’, and what is more, the *HEXOKINASE 3* (Prupe.1G366000) was strongly upregulated in the tolerant genotype, but consistently downregulated in the sensitive one during the whole hypoxia treatment (Figure 1) [70].

As a product of the phosphorylation of glucose by HXK, glucose 6-phosphate (G6P) can block sucrose-non-fermenting-related protein kinase-1 (SnRK1) activity [113]. SnRK1 is a kinase recognized as a metabolic sensor that can decode energy deficiency signals and induce the extensive metabolic reprogramming required for the adaptation to nutrient availability through inhibition of expensive energy processes and growth arrest. Stress conditions, such as hypoxia, can affect photosynthesis and photoassimilates biosynthesis along with respiration triggering a low energy syndrome (Tomé et al., 2014). Transcriptomics data evidenced the induction of *SnRK1* genes majorly associated with the hypoxia-sensitive *Prunus* rootstocks ‘Felinem’ [95] and ‘Mazzard F12/1’ (Figure 1, this review). Remarkably, low levels of G6P induce the activity of SnRK1, which triggers the signaling for upregulating several genes such as *PEPCKs*, commented on above. Regarding the genes encoding glycolytic enzymes, these two genotypes exhibited similar transcriptional activation between them, without significant differences in response to waterlogging. In grapevine, an upregulation of *DIPHOSPHATE-DEPENDENT PHOSPHOFRUCTOKINASES*, instead of *ATP DEPENDENT 6-PHOSPHOFRUCTOKINASES 1*, was found, responsible for converting D-fructose 6-phosphate to D-fructose 1,6-bisphosphate, which is another typical feature of the glycolysis in hypoxia that favors energy saving through PPi-dependent rather than ATP-dependent processes [41].

In order to maintain ATP production by the glycolytic pathway, it is necessary to replenish the NAD^+^ pool that was reduced during this process. In this context, activation of the fermentative pathways results in the classic and most widely reported response in the anaerobic metabolism. From pyruvate, lactic and ethanolic fermentation generate lactate and ethanol, respectively, and in the process NADH is oxidized, returning NAD^+^ to keep glycolysis active [68]. However, both fermentative pathways present eventual disadvantages, since lactate is toxic for the cells, and ethanol diffuses rapidly out of the cells, implying a considerable loss of carbon under hypoxic conditions [114]. The gene induction of *LACTATE DEHYDROGENASE* (*LDH*) in fruit trees under root hypoxia has been reported in avocado [94], *Prunus* spp. [70,112] and *C*. *sachalinensis* [53], but not in grapevine [41] or the *Prunus* genotypes analyzed by Rubio-Cabetas et al. [95]. In ‘Mariana 2624’ and ‘Mazzard F12/1’, root hypoxia triggered a rapid increase in the *LDH1* (Prupe.5 G072700) mRNA levels with a peak in their transcripts at six hours, but such increase was more dramatic and prolonged in the root hypoxia-sensitive genotype (Figure 1) [112]. Consequently, a higher L-lactate content was evidenced in ‘Mazzard F12/1’ roots with a maximum at 3 and 6 h of waterlogging. An increase in LDH activity in response to waterlogging in three species of *Prunus* (*P*. *mira*, *P*. *persica* and *P*. *amygdalus*) indicated that *P*. *amygdalus* was the genotype with a greater and more sustained increase in LDH activity. This fact was concomitant with its increased accumulation of lactic acid in the cytoplasm, and its lower tolerance to root hypoxia [115]. Lactate accumulation and cytoplasmic acidosis are determinants of hypoxia-sensitive phenotypes in maize [116]. In addition, in *Limonium* spp., plants capable of removing excesses of lactate from the cytoplasm were more tolerant to hypoxia conditions [117]. In this sense, the increase of *Prunus* spp. *NIP1;1* mRNA, a putative lactic acid transporter, was not linked to a lower lactate content in the roots of ‘Mazzard F12/1’. Bioinformatic approaches identified steric hindrances in PruavNIP1;1 given by the residues Phe107 and Trp88 in the NPA region and ar/R filter, respectively, but such blockages were absent in the NIP1;1 of ‘Mariana 2624’. The functional characterization of these aquaporins in the yeast strain Δjen1 corroborated the lower efficiency of the lactic acid transport of PruavNIP1;1, which could be related to a higher lactate accumulation and detrimental effects at cell level in ‘Mazzard F12/1’ roots under hypoxia [112].

The drop in cytoplasmic pH, in part related to the dissociation of lactic acid, inhibits LDH activity and stimulates that of pyruvate decarboxylase (PDC), the first step involved in ethanol fermentation [118]. *PDC* and *ALCOHOL DEHYDROGENASE* (ADH) transcripts were found to be expressed in each transcriptome analyzed. Both *PDC* and *ADH* transcripts were coordinately induced in ‘Mariana 2624’ and ‘Mazzard F12/1’ under O_2_ deficiency, although the hypoxia-tolerant genotype showed a decreasing trend after 6 h of waterlogging (Figure 1) [47]. This transcriptional finding suggests that ‘Mariana 2624’ resorts to other adaptation mechanisms, distinct to the fermentation pathways, after the first hours of flooding.

The pyruvate dehydrogenase complex catalyzes the oxidative decarboxylation of pyruvate with the formation of acetyl-CoA, CO_2_ and NADH(H^+^) [119], linking glycolysis to the Tricarboxylic acid (TCA) cycle. Interestingly, transcripts of the subunit E2 (*DIHYDROLIPOYL TRANS-ACETYLASE*, *DHLTA*), a component of this enzymatic complex, were downregulated in both ‘Mariana 2624’ and ‘Mazzard F12/1’ roots under hypoxic conditions; however, this repression was earlier and much stronger in the hypoxia-tolerant *Prunus* rootstock (Figure 1). This fact suggests a diminished metabolic flux in the TCA cycle and an accumulation of pyruvate. However, another conclusion for pyruvate as a result of O_2_ deficiency, different from the fermentative pathways, is its conversion to alanine by means of the enzyme ALANINE AMINOTRANSFERASE (AlaAT). The accumulation of this amino acid is typically linked to hypoxia in plants [114,120,121]. *AlaAT* mRNAs induced during hypoxia were reported in the roots of avocado [94], grapevine [41] and Myrobalan ‘P.2175’ [95]. Although *AlaAT* transcripts were not detected in the transcriptome of ‘Mariana 2624’ or ‘Mazzard F12/1’, the accumulation of alanine was evidenced in both genotypes during waterlogging, this being more intense in the hypoxia-sensitive genotype (Figure 1) [47]. A hypoxia-induced alanine accumulation is also possible through the activity of GABA-transaminase (GABA-T) that converts GABA into succinic semialdehyde (SSA), releasing alanine (from pyruvate) to the mitochondrial lumen [122,123]. Here, the eventual SSA accumulation will be toxic for the cell. The succinic-semialdehyde dehydrogenase (SSADH) converts SSA into succinate, consuming an NAD^+^ molecule [124] but this latter would limit the ability of the cell to properly maintain the active glycolysis.

Another alternative means of draining pyruvate to alanine by AlaAT under hypoxic conditions involves the glutamate metabolism. The reductive amination of 2-oxoglutarate by glutamine oxoglutarate amino transferase (GOGAT) regenerates glutamate (substrate of AlaAT to produce alanine) and NAD^+^ (Diab and Limami, 2016). Interestingly, root hypoxia induced *GOGAT* in ‘Mariana 2624’ rootstock, but not in the hypoxia-sensitive ‘Mazzard F12/1’, whose transcript levels remained unchanged under waterlogging (Figure 1). It is possible that a higher GOGAT enzyme activity is related to the higher metabolism of alanine in ‘Mariana 2624’, which would explain the modest accumulation of this amino acid during waterlogging in this genotype, as opposed to the notorious accumulation shown by the hypoxia-sensitive *Prunus* rootstock (Figure 1) [47]. In flooded grapevine, *GOGAT*, *GLUTAMINE SYNTHETASE* (*GS*) and *GLUTAMATE DEHYDROGENASE* (*GDH*) were overexpressed [41]. GS catalyzes the ATP-dependent assimilation of NH4^+^ into glutamine using glutamate as substrate. The GS/GOGAT cycle is the principal route of ammonium assimilation in plants [125]. In tomato (*Solanum lycopersicum* L.), the activity of the ATP-consuming GS was significantly enhanced in roots during prolonged root hypoxia [126]. Here, a striking contrast in the *GS* transcriptional pattern was evidenced between ‘Mariana 2624’ and ‘Mazzard F12/1’ roots under hypoxic conditions, as *GS* transcripts were strongly accumulated in the hypoxia-tolerant genotype after 24 h of waterlogging, but consistently downregulated as stress progressed in the hypoxia-sensitive one (Figure 1). This evidence suggests that a more active GS/GOGAT cycle, capable of assimilating nitrogen and regenerating NAD^+^ to support glycolytic flux under conditions of O_2_ deficiency, shapes one of the successful metabolic strategies involved in defining the hypoxia-tolerant phenotype seen in ‘Mariana 2624’. As in the flooded grapevine, *GDH* transcripts were overexpressed in the roots of waterlogged ‘Mazzard F12/1’, but clearly downregulated in the hypoxia-tolerant genotype (Figure 1). Another gene involved in the GABA shunt, *GLUTAMATE* *DECARBOXYLASE* (*GAD*), showed an upregulation only associated with the hypoxia-sensitive genotype (Figure 1). Thus, ‘Mazzard F12/1’ appears to boost the flux of the GABA shunt by acquiring glutamate from GDH-mediated 2-oxoglutarate amination (instead of from its inhibited GS/GOGAT cycle). This amination generates NAD^+^, but the detoxification of the SSA consequently generated in this pathway, through SSADH activity, consumes NAD^+^, so the net gain of this cofactor is 0.

The transcriptomic antecedents compiled from different fruit trees under hypoxic conditions show common alterations of genes involved in starch/sucrose metabolism and glycolysis, together with the inhibition of gluconeogenesis in the case of hypoxia-tolerant genotypes. The activation of, firstly, lactic fermentation (*LDH*), and then ethanolic fermentation (*PDC* and *ADH*), was also evident in all fruit trees. Since ethanol can represent a carbon leak from the plant, a more energy-efficient destination for pyruvate is its conversion to alanine. At this point, the regeneration of the glutamate involved in alanine biosynthesis from the GS/GOGAT cycle instead of from the GDH activity in the context of GABA shunt is postulated as one of the best metabolic strategies for explaining the survival and growth capacity during O_2_ deficiency in root hypoxia-tolerant genotypes, such as in the case of the *Prunus* rootstock ‘Mariana 2624’.

## 5. Root Respiration under O_2_ Deficiency

The carbon cycle is a bio-geochemical cycling process of the continuous flowing of organic and inorganic forms of carbon through the biosphere, geosphere and atmosphere, supporting life on Earth [127]. In the biosphere, plants play a key role via autotrophic respiration, which represents an important component of the carbon cycle and corresponds to respiratory processes in the leaf, shoot and root [128]. Respiration involves the participation of different processes responsible for the oxidation of glucose molecules for energy and carbon structures, either in the presence (aerobic) [129,130] or absence (anaerobic) of O_2_ [131]. Root respiration is a process sensitive to changes in soil conditions, such as chemical composition [132], temperature [133], salinity [134] and water excess (hypoxia/anoxia stress) [74], among others.

Root respiration is highly dependent on the availability of O_2_ in the root zone, and a lack of this element may lead plants into an imbalance in energy distribution within metabolic processes, whereby the deficit of ATP could range between 3% and 37.5% with respect to well-aerated roots [135]. When the O_2_ in the rhizosphere decreases to a point where the formation of ATP by cytochrome oxidase (COX, or complex IV) is hampered, the activation of less efficient metabolic pathways takes place (e.g., fermentative pathways) and plants may enter a state of energy crisis [136]. The growth and maintenance of tissues are two processes that require energy from root respiration, and must coexist coordinately for the correct development of plants. However, energy crisis caused by hypoxia/anoxia stress (O_2_ deficiency) induces an energy redistribution either to the maintenance or the growth of new tissue [74,137]. Membrane stability, active ion transport and de novo synthesis of proteins are the most expensive processes whereby the cell metabolism must adjust its energy budget [138]. It has been reported that these processes are controlled by gene regulation at both the transcript and translation level [139], and they strategically determine how plants cope with the energy reduction imposed by hypoxia/anoxia. It is well known that low O_2_ levels impair the respiratory metabolism of plant tissues. The damage induced by O_2_ depletion, especially on root respiration, could compromise the development and growth of the entire plant, because root respiration drives the energetic support for generating new biomass and/or cellular and structural maintenance [140,141,142].

**Figure 2:**
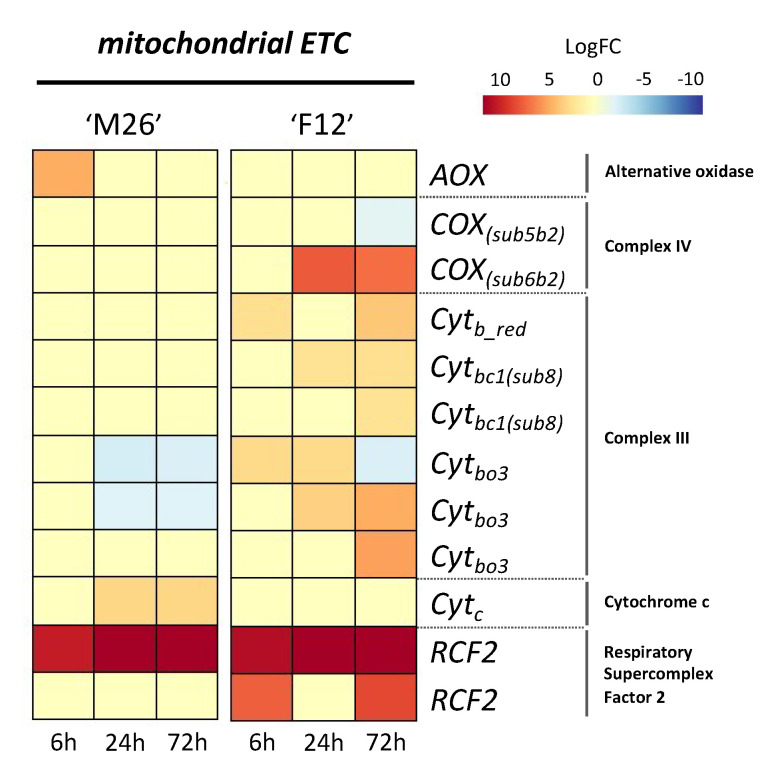
Transcriptomic reconfiguration of pathways involved in mitochondrial electron transport chain in response to O_2_ deficiency. The changes in the transcript levels of genes belonging to the mitochondrial electron transport chain typically related to energy metabolism, and that are expressed in a contrasting way between the genotypes, are described. Data obtained from de novo transcriptomic analysis of ‘Mariana 2624’ (‘M26′) and ‘Mazzard F12/1’ (‘F12′). *AOX*, alternative oxidase; *Cyt_c_*, Cytochrome *c*; *RCF2*, Respiratory Supercomplex Factor 2; *Cyt_bo3_*, Cytochrome bo3 ubiquinol oxidase; *Cyt_bc1(sub8)_*, Cytochrome bc1 complex subunit 8; *Cyt_b red_*, Cytochrome b reductase; *COX_(sub5b2)_*, Cytochrome c oxidase subunit 5b2; *COX_(sub6b2)_*, Cytochrome c oxidase subunit 5b2.

As previously commented, there is limited information available about transcriptome analysis in trees or woody species under low O_2_ conditions, and even fewer works have been reported that relate the differentiated expressions of genes from transcriptomes and the physiological responses of the respiratory metabolism. The respiratory chain has a principal function of transferring electrons to the terminal oxidases, where O_2_ acts as the final electron acceptor, producing high-energy phosphate bonds (ATP) [129,130]. The mitochondrial oxidative phosphorylation system consists of four multi-subunit oxidoreductases involved in the electron transport chain (mETC) (complexes I-IV) and the ATP synthase complex (complex V) [143,144]. In the revisited analysis of the transcriptomic study comparing *Prunus* rootstocks contrasting in their tolerance to hypoxia [70], DEGs encoding for proteins belonging to the mETC, such as *Respiratory Supercomplex Factor 2* (*RCF2*), subunits of complex III (*Cyt_bc1(sub8)_*, *Cyt_bo3_* and *Cyt_b red_*) and IV (*COX_(sub5b2)_* and *COX_(sub6b2)_*), *Cytochrome c* (*Cytc*) and *Alternative Oxidase* (*AOX*), were detected (Figure 2). The synchrony of the activity of each protein in the mETC may be altered depending on the O_2_ availability in the rhizosphere [145], having as a direct consequence a partially restricted or completely inhibited energy production [131]. As in herbaceous plants, in woody species one of the most dangerous subproducts of the aerobic metabolism is the formation of ROS such as H_2_O_2_ and O_2_^−^ [62]; however, under an anaerobic condition, other harmful molecules are also formed. Thus, the combination of ROS and nitric oxide (NO) may be extremely detrimental for the cell [70,146].

**Figure 3:**
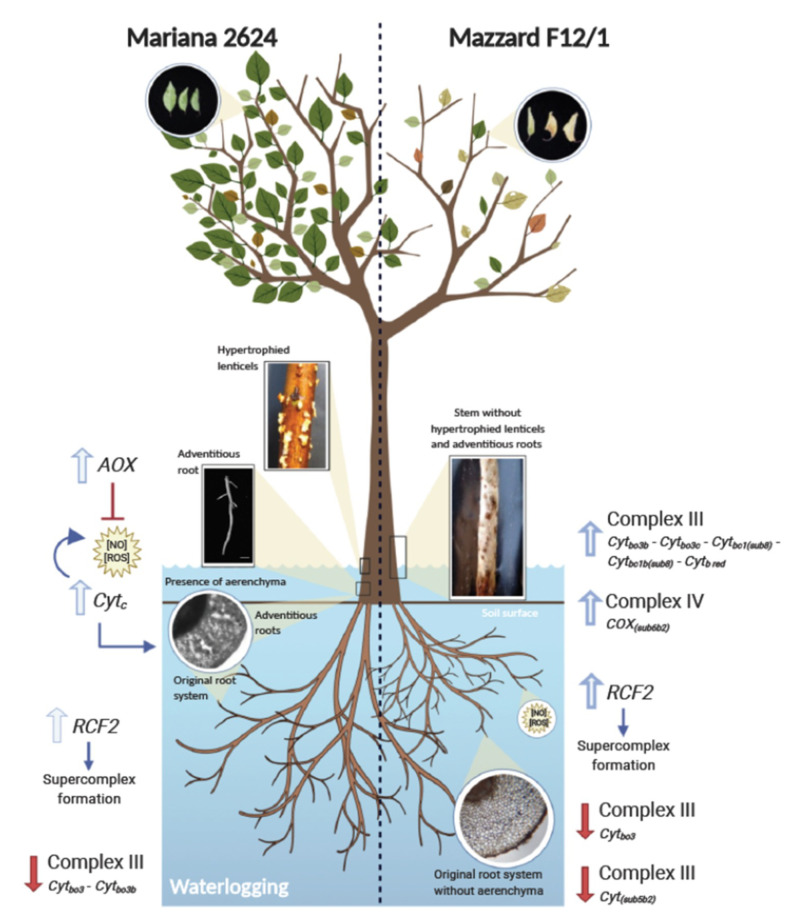
Schematic overview of principal mETC-expressing genes and morpho-anatomical changes in *Prunus* rootstocks under root hypoxia. The figure summarizes the effect of *AOX* and *Cyt_c_* on NO and ROS generation. Regarding ROS, the early induction of *AOX* (6 h) and the late induction of *Cyt_c_* (24 and 72 h) would lead to a regulation of ROS accumulation in a temporally non-exclusive way in the root-hypoxia-tolerant *Prunus* rootstock (‘Mariana M2624’). Thus, at the beginning of stress by O_2_ deficiency, this genotype would be preventing the accumulation of ROS in roots, but as stress progresses, in the context of apoptosis, the elevation of ROS levels has been associated with the activity of Cyt_c_ [171,172]. The generation of aerenchyma mediated by apoptosis present in the original root system, and the generation of adventitious roots with aerenchyma and hypertrophied lenticels, are characteristic features of ‘Mariana M2624’. These traits are completely absent in the root-hypoxia-sensitive *Prunus* rootstock (‘Mazzard F12/1’). The generation of air-filled spaces results in an avoidance strategy in relation to O_2_ deprivation, which is associated with the maintenance of an adequate metabolism and energy supply at the whole plant level. The blue and red arrows represent the over- and down-expression of genes related to mETC. *AOX*, alternative oxidase; *Cyt_c_*, Cytochrome *c*; *RCF2*, Respiratory Supercomplex Factor 2; *Cyt_bo3_*, Cytochrome bo3 ubiquinol oxidase; *Cyt_bc1(sub8)_*, Cytochrome bc1 complex subunit 8; *Cyt_b red_*, Cytochrome b reductase; *COX_(sub5b2)_*, Cytochrome c oxidase subunit 5b2; *COX_(sub6b2)_*, Cytochrome c oxidase subunit 5b2; [NO], nitric oxide; [ROS], reactive oxygen species. Images of morpho-anatomical changes were obtained from [7,74]. This figure was created using BioRender.

The processes involved in coping with low O_2_ at the root level are quite expensive for the plant’s energy budget [137,147], and therefore to scavenge harmful molecules, plants are required to invest a significant amount of energy in synthesizing expensive enzymatic or nonenzymatic molecule scavengers [148]. In the mETC, ROS are formed principally through electron leakage from the protein complexes inserted into the mitochondrial membrane, such as complex I (NDH, NADH dehydrogenase) and III (Cyt*_bc1_*, cytochrome bc1 dehydrogenase) [149], and NO formation is more associated with alternative oxidase (*AOX*), complex III and IV (*COX*, cytochrome *c* oxidase) [150,151,152]. The revisited analysis of [70] revealed large differences in gene expression related to mETC, which are closely related to proteins of complex III, IV and *AOX* (Figure 2). Regarding complex III, no alterations were found in the expressions of the *Cyt_b_red_* and *Cyt_bc1_* genes in waterlogged ‘Mariana 2624’ plants, however the gene inductions of these isoforms were evident in the root-hypoxia-sensitive genotype ‘Mazzard F12/1’ in response to O_2_ deficiency (Figure 2). On the other hand, the root-hypoxia-tolerant ‘Mariana 2624’ repressed the expression of two *Cyt_bo3_* isoforms, but ‘Mazzard F12/1’ showed a different behavior since the three *Cyt_bo3_* isoforms were induced in response to hypoxia in both early and late stages (Figure 2). With respect to complex IV, ‘Mariana 2624’ did not present DEGs over time, while in contrast, ‘Mazzard F12/1’ reduced the expression of *COX_(sub5b2)_* at 72 h, but a strong induction of *COX_(sub6b2)_* was evident from 24 h of root hypoxia (Figure 2). Some of the protein complexes of the mitochondrial membrane may contribute to the scavenging of these harmful molecules. In this sense, NO is formed from nitrite by COX, however, this protein is inhibited by the raised NO, while that *AOX* is an NO-resistant protein [153]. For instance, it has been found in *A. thaliana* that the overexpression of *AOX* may prevent excesses of NO modulating the formation of ONOO^−^ from interacting with O_2_^−^ [153]. Therefore, despite the low affinity of O_2_ with *AOX* and the limiting proton translocation, the activity of *AOX* allows the maintenance of the energy balance under hypoxic conditions [154]. Recently, Vishwakarma et al. [153] found an increase in the haemoglobin–nitric oxide (Hb/NO) cycle under hypoxia, which might be mediated by the *AOX* protein and improves the redox and energy status of the hypoxic cell [136]. This cycle consumes NADH-regenerating NAD^+^, which would contribute to maintaining the glycotytic flux during O_2_ deficiency [155]. In the roots of *Prunus* rootstock under O_2_ deficiency, the class 1 *non-symbiotic haemoglobin*-like (*nsHb*) gene showed a higher expression in the hypoxia-tolerant genotype than in the sensitive one [156]. This was also found in roots of hypoxia-tolerant oak genotypes under low O_2_ [157]. In addition, the transcriptomic analysis revealed a higher *AOX* gene expression in ‘Mariana2624’ at 6 h of waterlogging, but no upregulation of this gene was detected in the sensitive genotype (Figure 2 and Figure 3). Thereby, the participation of class 1 *nsHb* and *AOX* genes in hypoxia tolerance genotypes may suggest that the possibility of the participation of the nsHb/NO cycle drives energetic support to the roots of woody plants in the early stages of hypoxia, promoting the electron flow [158] and allowing the prevention of mETC overreduction [159].

To generate an optimal response under hypoxia is necessary in order to activate the complete genetic machinery, which could have an extra cost to the root metabolism [160]. Under hypoxia, the control of protein synthesis may be a ‘double-edged sword’, since this is necessary for proper cell function, but an increase in protein turnover may increase the respiratory costs of maintenance, which might lead to compromised growth [138]. Under optimal conditions, the energy generated in mitochondrial phosphorylation as ATP is used to synthesize new structures in growing plants (growth respiration), and for all processes related to cellular maintenance, such as protein turnover, maintenance of ion gradients and membrane potentials in the cell (maintenance respiration) [141,142,161]. However, environmental changes may alter the distribution of energy. Under waterlogging, the forced entry into a state of energy crisis leads the roots to allocate resources to priority processes, thus modifying the costs associated with root respiration [138]. In *Carex* plants, O_2_ deficiency drives the activation of strategies for reducing root growth in order to maximize the respiratory cost imposed on I upon uptake [137]. However, the allocation of O_2_ to components of root respiration depends on the species. In the hypoxia-tolerant *Prunus* genotype, ‘Mariana M2624’, Toro et al. [74] found a greater ability of the root growth to spend less energy in processes related to maintenance, such as protein turnover and membrane integrity. These costs could reach up to 80% of the plant’s energy budget [162], and therefore its regulation would be a key factor in tolerance to hypoxia. An EuKaryotic Orthologous Groups (KOG) classification in *Cerasus sachalinensis* roots under short-term waterlogging showed that the largest groups of DEGs were included in the categories of post-translational modification, protein turnover, and chaperones [53]. In addition, the authors found that a high number of transcripts were associated with translation pathways, and also energy metabolism. The revisited transcriptomic analysis showed a remarkable difference between transcripts related to mETC from ‘Mariana 2624’ and ‘Mazzard F12/1’ rootstocks in response to hypoxia. Thus, low O_2_ in roots increases dramatically the gene expression of the sensitive genotype from 6 to 72 h of waterlogging, whereat one would usually find upregulated genes from subunits of complex III (*Cyt_bo3_*, *Cyt_b_red_*, and *Cyt_bc1_*) and IV (*COX_(sub6b2)_*), and genes that have control over processes associated with supercomplex formation (*RCF2*) (Figure 2 and Figure 3). According to Arru and Fornaciari [160], protein synthesis would depend on post-transcriptional or post-translational regulation, there being extremely high energy-requiring step at the translational level. A study performed on *Prunus* showed that hypoxia-tolerant rootstocks manifested reduced ATP demands for protein turnover and the maintenance of membrane integrity, which was closely related to the low respiratory costs of maintenance [74].

Into the mitochondrial inner membrane, the respiratory protein complexes associate to form supramolecular assemblies known as supercomplexes [163,164]. The supercomplex formation seems to be crucial for the proper functioning of mETC, and requires the assistance of specific genes to efficiently assemble its constituent proteins [165]. In higher plants, the presence of the supercomplex has been identified in several species such as *Arabidopsis*, bean, potato and barley [164]. However, there is still a lack of information about the genes encoding for proteins involved in supercomplex formation. The *Respiratory Supercomplex Factor 2* (*RCF2*) gene is part of the conserved gene family termed *hypoxia-induced gene 1*, which is highly expressed under hypoxia conditions and has been described as necessary for supercomplex formation [166,167].

As for other genes from mETC, there are scarce reports about the *RCF2* genes of woody species or even of higher plants. Recently, Shin et al. [168] identified the *RCF2* gene from *Cucumis melo*, but further efforts are required to identify this gene in a larger number of woody species and evaluate its response under O_2_ deficiency. Further, in wheat roots, *RCF2* helps to overcome an energy deficit by enhancing ADP/ATP transfer and, ultimately, improving the supply of ATP [158]. An early expression of the *RCF2* gene in both hypoxia-tolerant and -sensitive *Prunus* rootstocks is evidenced (Figure 2 and Figure 3). However, only the hypoxia-tolerant genotype ‘Mariana 2624’ showed expression of *AOX*, while the hypoxia-sensitive genotype ‘Mazzard F12/1’ showed the expression of genes related to complex III, and after 72 h also evidenced the expression of genes related to complex IV (Figure 2). According to Eubel et al. [164], in the mETC, complex III is commonly found, forming a higher (I+III) and lower (III+IV) abundance of the supercomplex, and on the other hand, *AOX* does not seem to form part of a supercomplex, because complex I and III would limit *AOX* activity by reducing substrate ubiquinol. It is has been reported that higher levels of complex I and supercomplex I+III could contribute directly to the maintenance of mitochondrial function under hypoxia [152]. The transcriptomic data of mETC genes from RNAseq could lead to the proposal that the presence of the AOX gene (and consequently AOX protein) would not be limited by the presence of genes related to complex III, which could lead to an over-formation of the supercomplex and reduce the AOX protein activity (Figure 2). In addition, the fact that the ‘Mariana 2624’ rootstock did not express genes related to complex III or IV, although *RCF2* genes were effectively expressed, would indicate an appropriate regulation of the supercomplex in the mETC in the tolerant genotype. In the hypoxia-sensitive *Prunus* rootstock ‘Mazzard F12/1’, a higher energy cost related to protein turnover was reported as being triggered by O_2_ deficiency, and root tissue injury triggered by waterlogging [74]. As consequence, the hypoxia-sensitive genotype should require greater protein synthesis/breakdown, which is partially observed here in the high differential expression of the complex proteins at different times of waterlogging (Figure 2). mETC have several protein complexes with different roles that guarantee the maintenance of cellular energy both under optimal and stress conditions. Cytochrome c (*Cyt_c_*) corresponds to a small and conserved protein family that is responsible for generating the proton gradient across complexes III and IV, driving ATP synthesis [169] and in addition potentially plays a key role in the development of an adaptative mechanism for tolerating low O_2_ [170]. It has been described that the *Cyt_c_* protein may be released (with ROS production) from the mitochondrial membrane into the cytosol, to trigger the key step in the early execution phase of programmed cell death (apoptosis) [171,172].

Generally, the avoiding strategies of plant adaptation to O_2_ deficiency involve the formation of aerenchyma structures by apoptosis, in order to support the increase in O_2_ diffusion and maintain the aerobic energy supply in root cells [7,74,80]. Scarce information for woody species has been reported regarding the relationship between *Cyt_c_* and hypoxia stress [170]; however, it is widely known that waterlogged woody species are able to develop aerenchyma in the roots through apoptosis [5,7,74,173]. The *Cyt_c_*-dependent aerenchyma formation relies on the redox state of the cell environment, which will depend on the presence of H_2_O_2_ and/or O_2_^-^ [174]. Under hypoxia, there are relatively high concentrations of H_2_O_2_ [175], which would lead the cellular environment to an oxidized state, changing the *Cyt_c_* protein structure into its oxidized state, which is capable of triggering apoptosis [174]. After 24 h of waterlogging treatment, a higher induction of the *Cyt_c_* gene was detected in the hypoxia-tolerant genotype ‘Mariana 2624’, while no or very low expression was observed in ‘Mazzard F12/1’ (Figure 2 and Figure 3). On the other hand, waterlogged ‘Mariana 2624’ plants developed aerenchyma in roots, which would combine with the air-filled spaces in supplying the O_2_ needed for aerobic metabolic processes [7,74] (Figure 3). Therefore, we suggest that under low O_2_ stress, the overexpression of the *Cyt_c_* gene in an oxidized cell environment could increase the synthesis of *Cyt_c_* proteins, generating a higher concentration of proteins in the cytoplasm, which would support the induction of apoptosis and end with aerenchyma formation (Figure 3). Certainly, in the future, more comprehensive studies with woody plants are required to help understand the steps between *Cyt_c_* gene expression and the modulation of protein synthesis, especially under conditions of O_2_ deficiency.

## 6. Conclusions and Perspectives in Fruit Trees Research

The effects of oxygen deficiency and the adaptive responses of plants have been extensively studied in herbaceous species, mainly with annual life cycles. However, woody fruit tree species, of great economic importance in temperate and sub-tropical zones, have been poorly attended. From a physiological point of view, characterizing the adjustment of photosynthesis to hypoxic conditions is a useful approach to contribute to the definition of the tolerance of these species to such environmental stress. Thus, species more tolerant to root hypoxia, capable of maintaining higher levels of photosynthetic activity, may provide greater carbohydrate reserves for facing the energy crisis triggered by anaerobiosis. On the other hand, transcriptomic studies have become useful for getting a broader view of the metabolic adaptation of fruit trees to hypoxia. Furthermore, together with evaluating the classically described metabolic pathways for plants under hypoxia, transcriptomic analyses allow the investigation of routes or processes less explored in these perennial plant species, such as the genetic determinants of energy sensing or the genes involved in the mETC.

In this review, we focused on analyzing the physiological and molecular aspects of the responses of fruit trees under root hypoxia, with emphasis on a study model that involves two genotypes of *Prunus* rootstocks with contrasting tolerances to O_2_ deficiency. In light of the multiple and diverse antecedents evaluated, it seems clear that the tolerant species of fruit trees manage to adapt and survive waterlogging or flooding due to their ability to detect oxygen deficiency more quickly and change their metabolism through a suitable transcriptomic reprogramming.

In this sense, keeping calm, and avoiding the activation of more routes than those that are strictly necessary or less energy efficient in an anaerobic environment, would allow the plant to invest its energy budget precisely, without exhausting it. This would be exemplified by the measured amount of DEGs detected in the root-hypoxia-tolerant *Prunus* rootstock versus the massive transcriptomic reconfiguration evidenced in the sensitive genotype ‘Mazzard F12/1’, implying a higher energy expenditure for the latter. At the same time, the anatomical and biochemical factors that operate in favor of maintenance processes, which are less energy demanding than those of repair, contribute to the early adaptation to root hypoxia of the tolerant genotype. Subsequently, late morpho-anatomical modifications end up defining a hypoxia-avoidance strategy that allows long-term survival in tolerant genotypes.

Finally, further studies are needed into the effects that re-oxygenation exerts on the fruit trees during the hypoxia recovery phase. In addition, a rising challenge in the study of the adaptive response to root hypoxia of woody fruit trees would involve epigenetic approaches oriented towards analyzing the phenomena of the memory of stress that can be transmitted between rootstocks and scion, or between growing seasons, and how it affects the yielding behavior of the orchards.

## Figures and Tables

**Table 1:** Transcript levels (of genes related to ROS production (*RBOH*) and ROS scavenging (*SOD*, *CAT* and *APX*)) from transcriptomics data from *Prunus* rootstocks under hypoxia.

		Log_2_ FC					
		‘Mariana 2624’			‘Mazzard F12/1’		
Gene		6 h	24 h	72 h	6 h	24 h	72 h
*RBOHA*	Prupe.6G321500	4.751	4.196	3.828	3.940	3.656	3.112
*RBOHC/RHD2*	Prupe.1G211000	−0.300	−1.902	−1.240	0.944	0.935	1.138
*RBOHE*	Prupe.5G107400	0.398	0.309	0.618	0.224	0.429	0.770
*Cu Zn SOD1*	Prupe.2G269400	−0.451	−1.268	−2.900	−0.454	−0.695	−0.863
*Cu Zn SOD2*	Prupe.1G347200	0.911	2.752	2.557	−0.713	−1.446	−0.485
*Cu Zn SOD3*	Prupe.2G262400	1.022	3.112	2.748	0.930	3.460	2.933
*Fe SOD1*	Prupe.6G042300	0.512	1.369	0.869	1.058	1.402	0.840
*CAT1*	Prupe.5G011300	0.174	−0.134	−0.818	nd	nd	nd
*CAT2*	Prupe.5G011400	0.498	−0.115	−0.526	0.376	−0.739	−0.580
*APX 1*	Prupe.1G481000	0.681	0.724	0.792	0.685	0.873	0.525
*APX 2*	Prupe.1G493900	−0.383	−0.638	−0.547	−0.017	−0.156	−0.395
*APX 3*	Prupe.6G091600	0.719	−1.498	−2.527	0.555	−1.351	−2.630
*APX 5*	Prupe.6G242200	6.538	7.284	6.864	3.323	3.948	4.591
*APX 6*	Prupe.7G171200	0.718	0.106	−0.252	1.158	0.502	0.740
*APX S*	Prupe.8G164400	−0.421	−2.284	−2.494	−0.414	−1.564	−2.650

FC: fold change; nd: no detected value.
